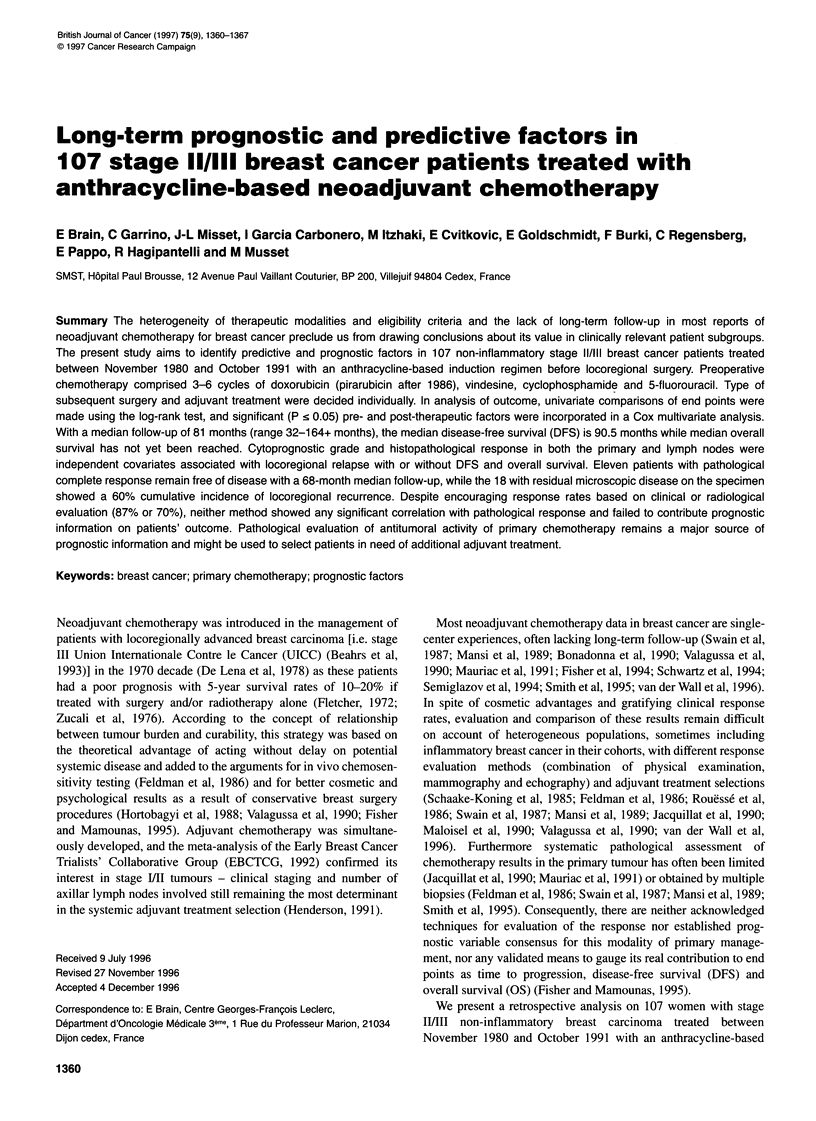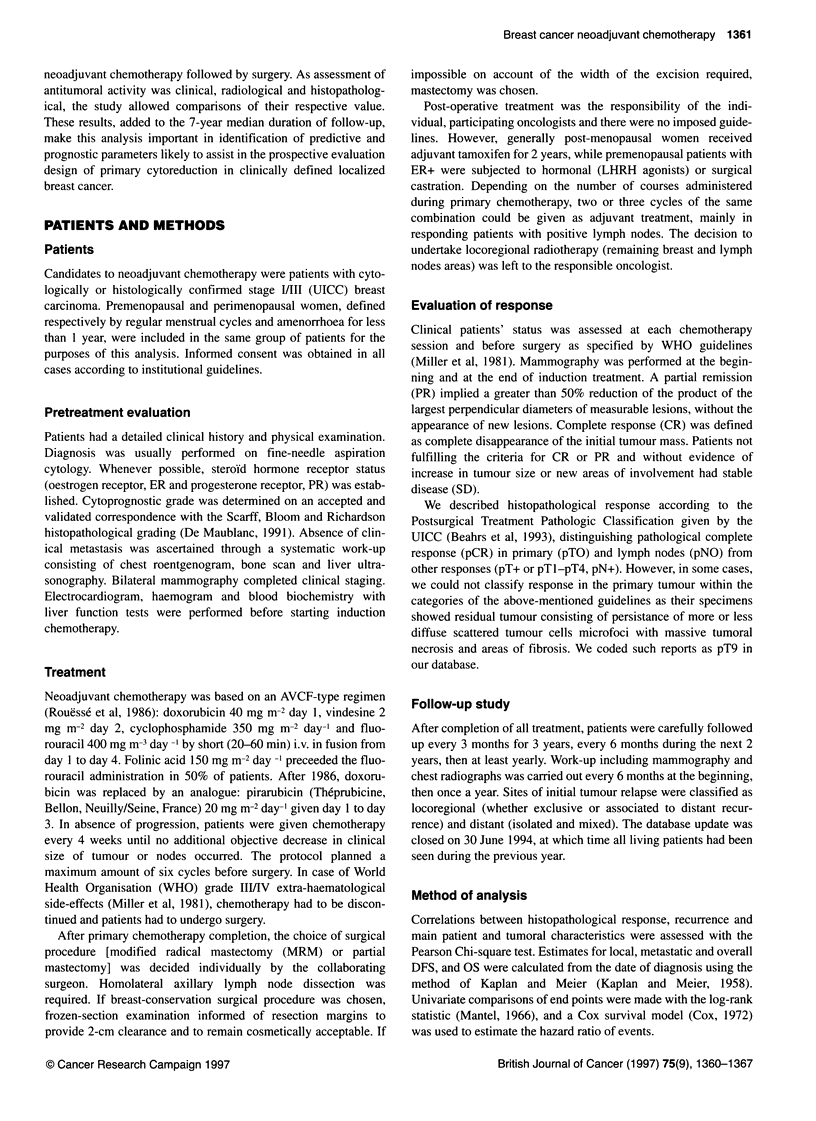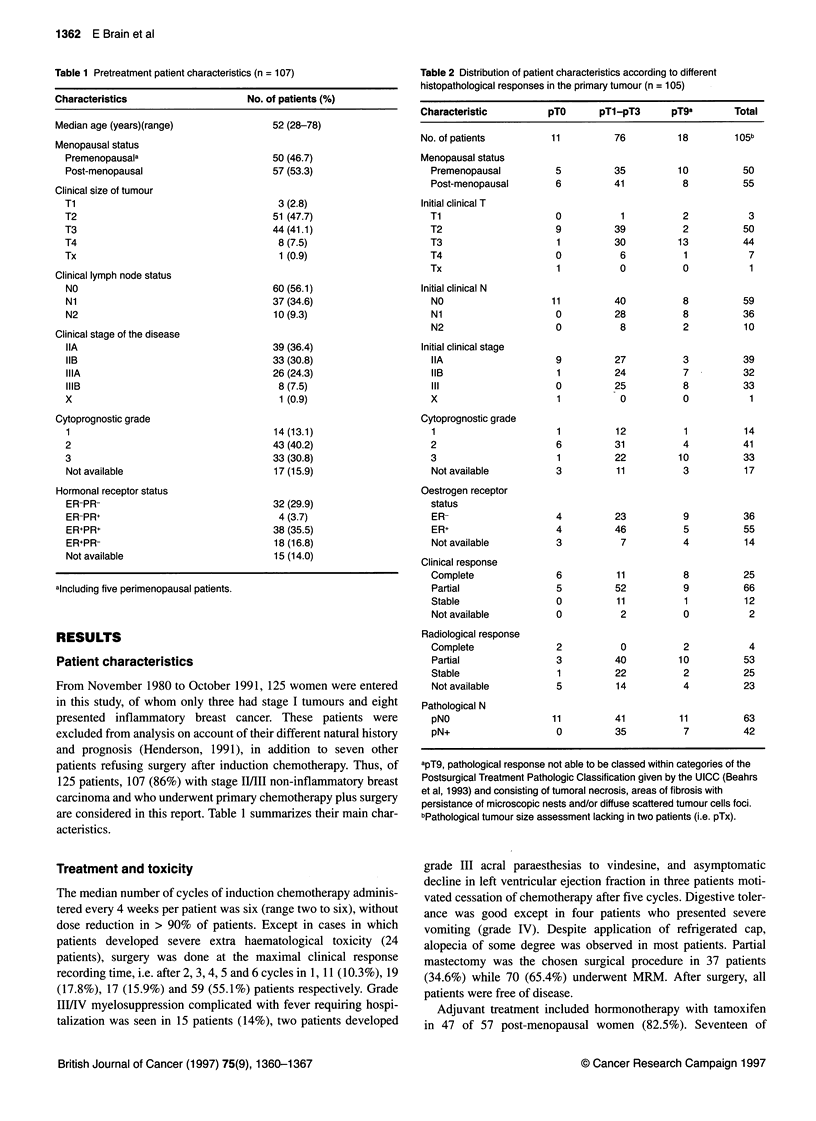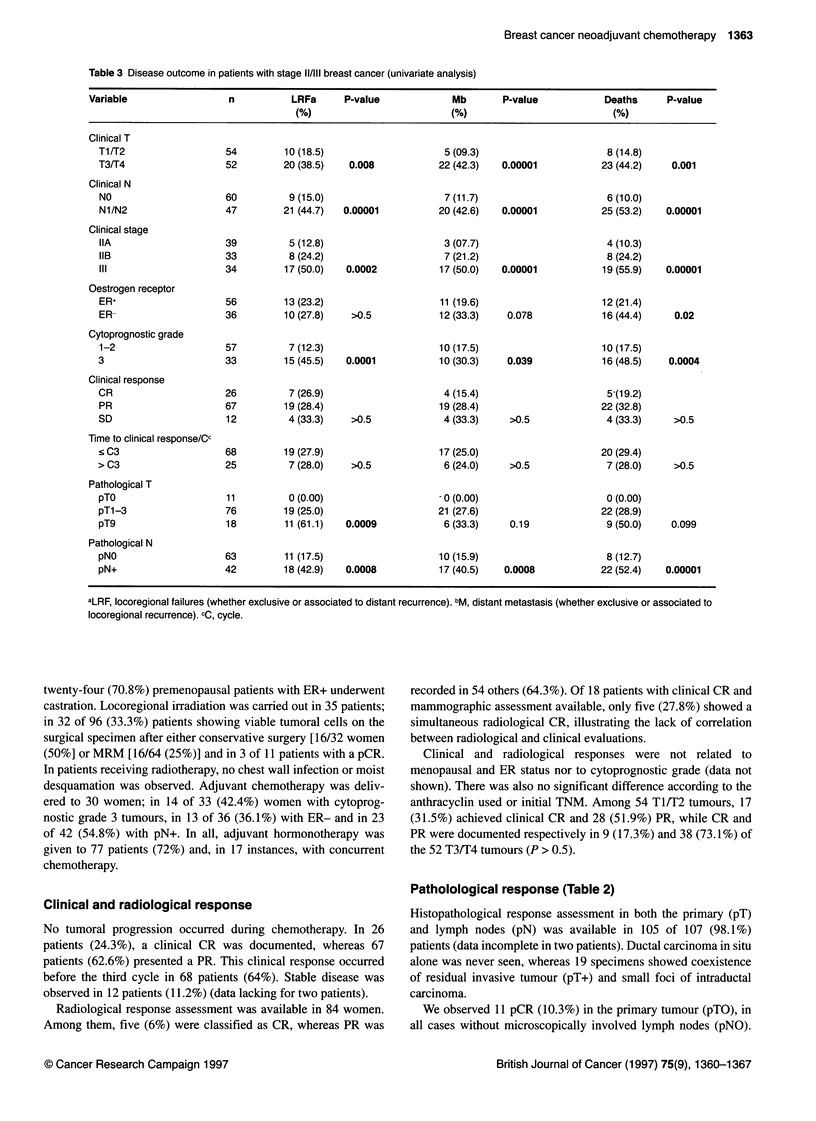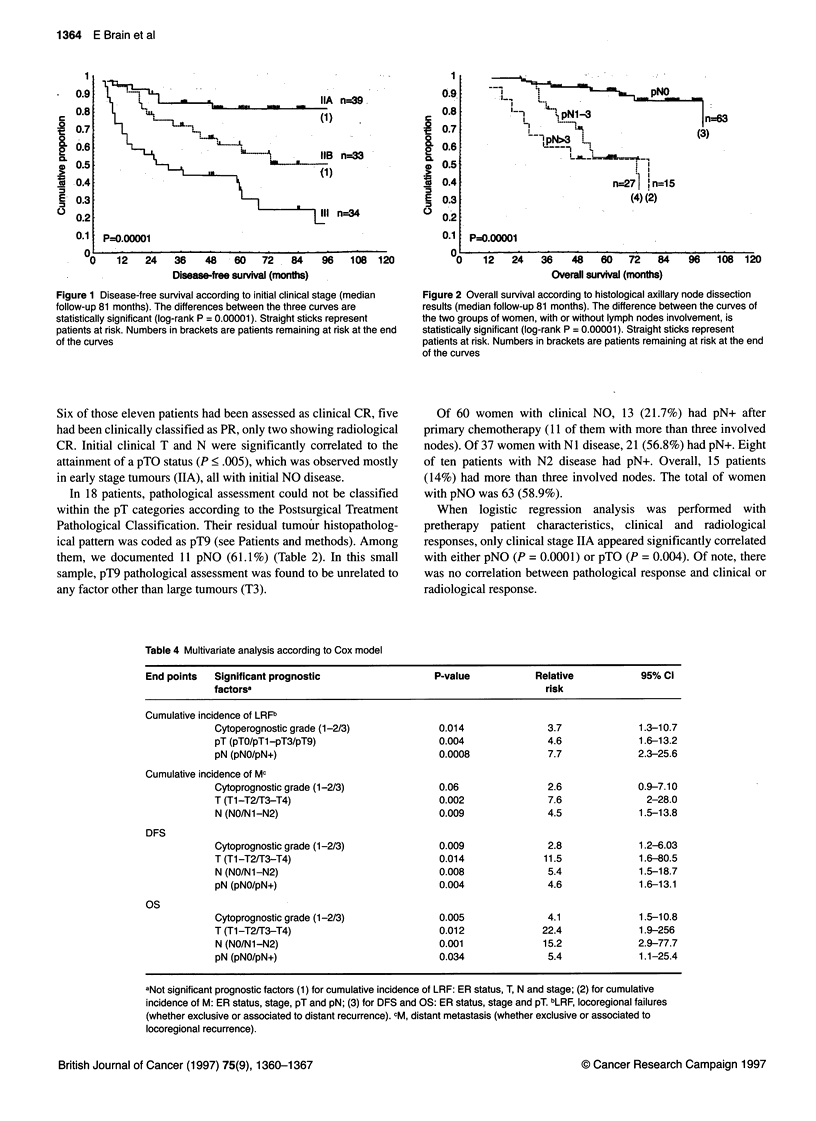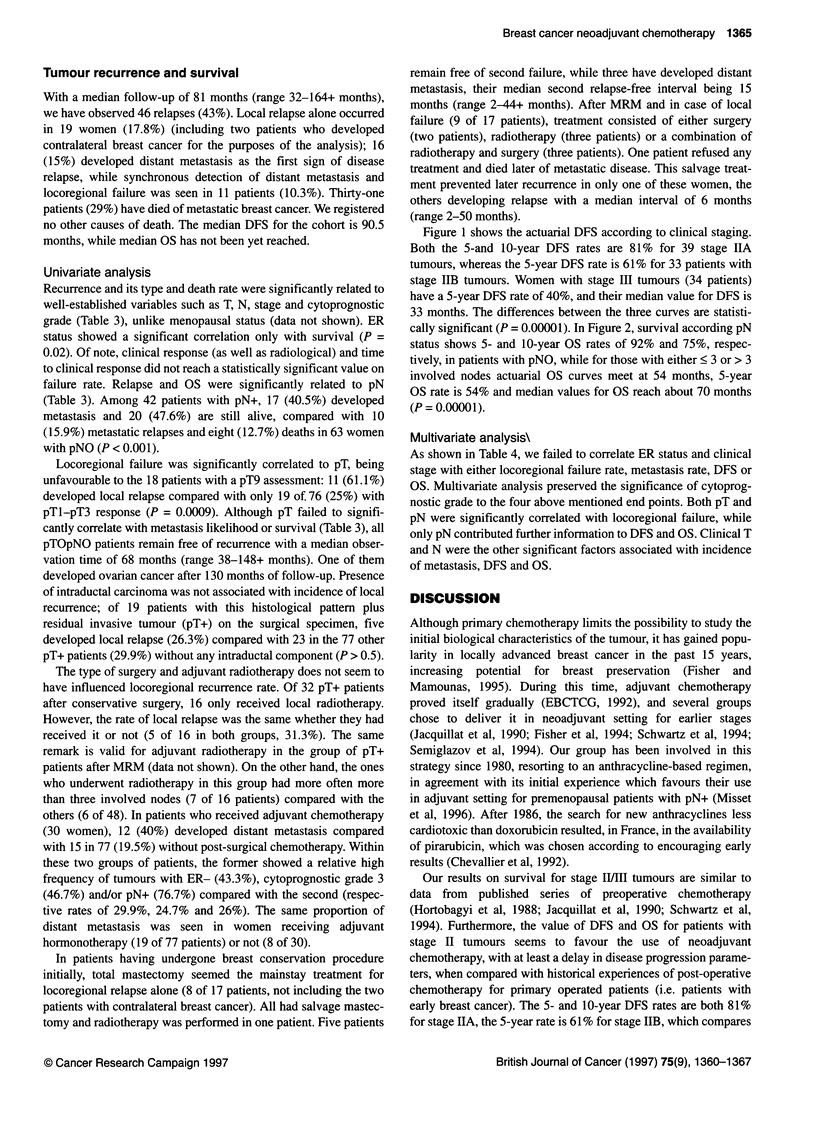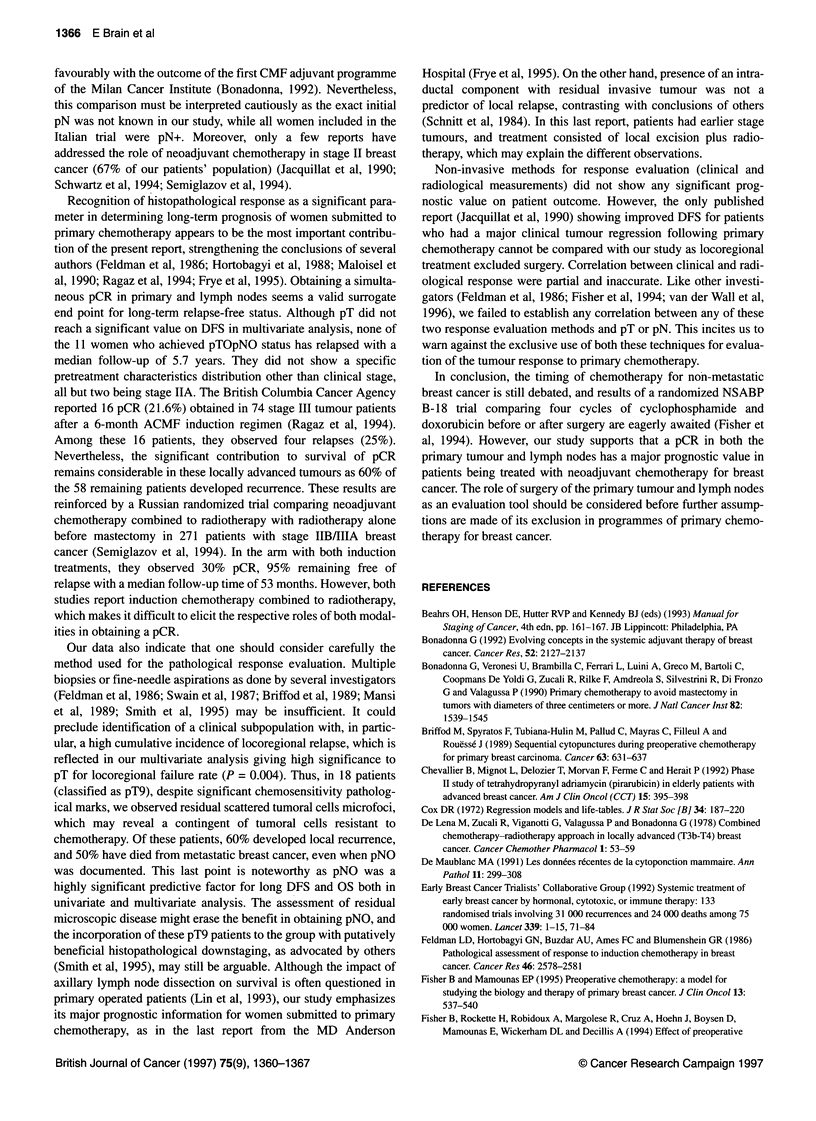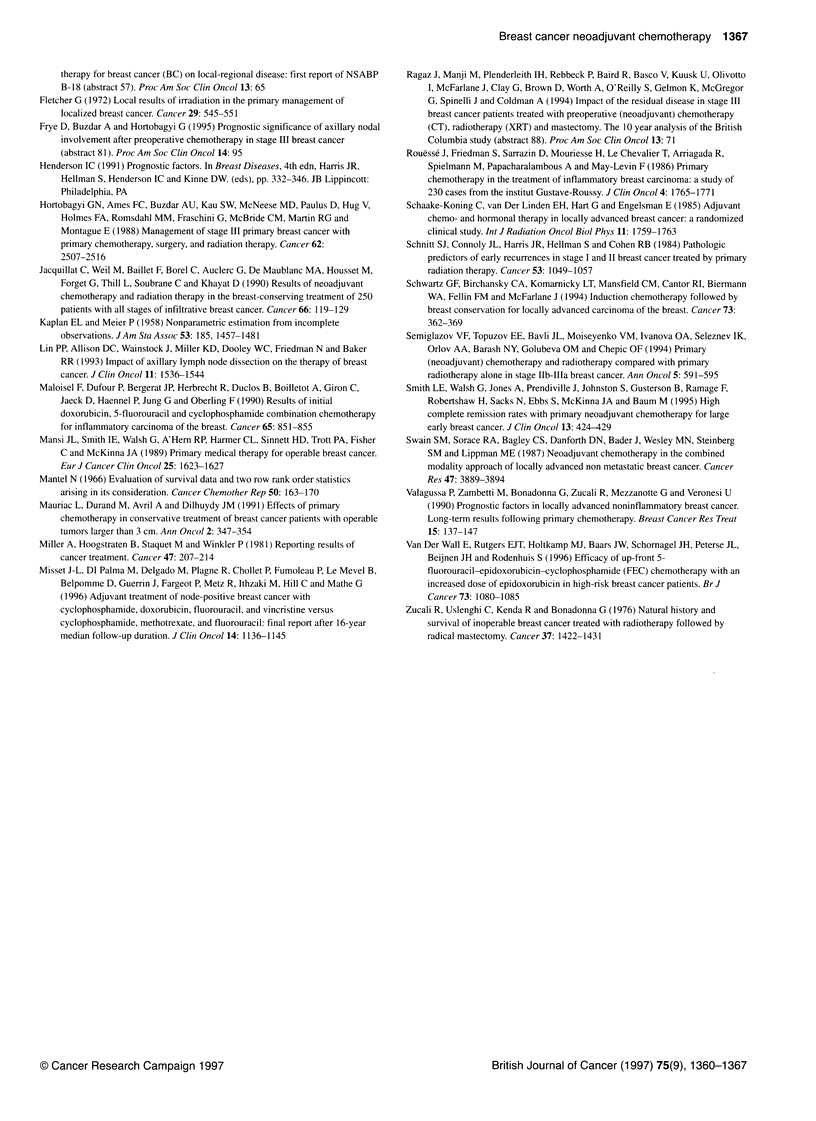# Long-term prognostic and predictive factors in 107 stage II/III breast cancer patients treated with anthracycline-based neoadjuvant chemotherapy.

**DOI:** 10.1038/bjc.1997.230

**Published:** 1997

**Authors:** E. Brain, C. Garrino, J. L. Misset, I. G. Carbonero, M. Itzhaki, E. Cvitkovic, E. Goldschmidt, F. Burki, C. Regensberg, E. Pappo, R. Hagipantelli, M. Musset

**Affiliations:** SMST, HÃ´pital Paul Brousse, Villejuif, France.

## Abstract

The heterogeneity of therapeutic modalities and eligibility criteria and the lack of long-term follow-up in most reports of neoadjuvant chemotherapy for breast cancer preclude us from drawing conclusions about its value in clinically relevant patient subgroups. The present study aims to identify predictive and prognostic factors in 107 non-inflammatory stage II/III breast cancer patients treated between November 1980 and October 1991 with an anthracycline-based induction regimen before locoregional surgery. Preoperative chemotherapy comprised 3-6 cycles of doxorubicin (pirarubicin after 1986), vindesine, cyclophosphamide and 5-fluorouracil. Type of subsequent surgery and adjuvant treatment were decided individually. In analysis of outcome, univariate comparisons of end points were made using the log-rank test, and significant (P < or = 0.05) pre- and post-therapeutic factors were incorporated in a Cox multivariate analysis. With a median follow-up of 81 months (range 32-164+ months), the median disease-free survival (DFS) is 90.5 months while median overall survival has not yet been reached. Cytoprognostic grade and histopathological response in both the primary and lymph nodes were independent covariates associated with locoregional relapse with or without DFS and overall survival. Eleven patients with pathological complete response remain free of disease with a 68-month median follow-up, while the 18 with residual microscopic disease on the specimen showed a 60% cumulative incidence of locoregional recurrence. Despite encouraging response rates based on clinical or radiological evaluation (87% or 70%), neither method showed any significant correlation with pathological response and failed to contribute prognostic information on patients' outcome. Pathological evaluation of antitumoral activity of primary chemotherapy remains a major source of prognostic information and might be used to select patients in need of additional adjuvant treatment.


British Journal of Cancer (1997) 75(9), 1360–1367
© 1997 Cancer Research Campaign

### Long-term prognostic and predictive factors in 107 stage II/III breast cancer patients treated with anthracycline-based neoadjuvant chemotherapy

**E Brain, C Garrino, J-L Misset, I Garcia Carbonero, M Itzhaki, E Cvitkovic, E Goldschmidt, F Burki, C Regensberg, E Pappo, R Hagipantelli and M Musset**

SMST, Hôpital Paul Brousse, 12 Avenue Paul Vaillant Couturier, BP 200, Villejuif 94804 Cedex, France

**Summary** The heterogeneity of therapeutic modalities and eligibility criteria and the lack of long-term follow-up in most reports of neoadjuvant chemotherapy for breast cancer preclude us from drawing conclusions about its value in clinically relevant patient subgroups. The present study aims to identify predictive and prognostic factors in 107 non-inflammatory stage II/III breast cancer patients treated between November 1980 and October 1991 with an anthracycline-based induction regimen before locoregional surgery. Preoperative chemotherapy comprised 3–6 cycles of doxorubicin (pirarubicin after 1986), vindesine, cyclophosphamide and 5-fluorouracil. Type of subsequent surgery and adjuvant treatment were decided individually. In analysis of outcome, univariate comparisons of end points were made using the log-rank test, and significant ($P \leq 0.05$) pre- and post-therapeutic factors were incorporated in a Cox multivariate analysis. With a median follow-up of 81 months (range 32–164+ months), the median disease-free survival (DFS) is 90.5 months while median overall survival has not yet been reached. Cytoprognostic grade and histopathological response in both the primary and lymph nodes were independent covariates associated with locoregional relapse with or without DFS and overall survival. Eleven patients with pathological complete response remain free of disease with a 68-month median follow-up, while the 18 with residual microscopic disease on the specimen showed a 60% cumulative incidence of locoregional recurrence. Despite encouraging response rates based on clinical or radiological evaluation (87% or 70%), neither method showed any significant correlation with pathological response and failed to contribute prognostic information on patients' outcome. Pathological evaluation of antitumoral activity of primary chemotherapy remains a major source of prognostic information and might be used to select patients in need of additional adjuvant treatment.

**Keywords:** breast cancer; primary chemotherapy; prognostic factors

Neoadjuvant chemotherapy was introduced in the management of patients with locoregionally advanced breast carcinoma [i.e. stage III Union Internationale Contre le Cancer (UICC) (Beahrs et al, 1993)] in the 1970 decade (De Lena et al, 1978) as these patients had a poor prognosis with 5-year survival rates of 10–20% if treated with surgery and/or radiotherapy alone (Fletcher, 1972; Zucali et al, 1976). According to the concept of relationship between tumour burden and curability, this strategy was based on the theoretical advantage of acting without delay on potential systemic disease and added to the arguments for in vivo chemosensitivity testing (Feldman et al, 1986) and for better cosmetic and psychological results as a result of conservative breast surgery procedures (Hortobagyi et al, 1988; Valagussa et al, 1990; Fisher and Mamounas, 1995). Adjuvant chemotherapy was simultaneously developed, and the meta-analysis of the Early Breast Cancer Trialists' Collaborative Group (EBCTCG, 1992) confirmed its interest in stage I/II tumours – clinical staging and number of axillar lymph nodes involved still remaining the most determinant in the systemic adjuvant treatment selection (Henderson, 1991).

Received 9 July 1996
Revised 27 November 1996
Accepted 4 December 1996

Correspondence to: E Brain, Centre Georges-François Leclerc, Départment d'Oncologie Médicale 3ème, 1 Rue du Professeur Marion, 21034 Dijon cedex, France

Most neoadjuvant chemotherapy data in breast cancer are single-center experiences, often lacking long-term follow-up (Swain et al, 1987; Mansi et al, 1989; Bonadonna et al, 1990; Valagussa et al, 1990; Mauriac et al, 1991; Fisher et al, 1994; Schwartz et al, 1994; Semiglazov et al, 1994; Smith et al, 1995; van der Wall et al, 1996). In spite of cosmetic advantages and gratifying clinical response rates, evaluation and comparison of these results remain difficult on account of heterogeneous populations, sometimes including inflammatory breast cancer in their cohorts, with different response evaluation methods (combination of physical examination, mammography and echography) and adjuvant treatment selections (Schaake-Koning et al, 1985; Feldman et al, 1986; Rouëssé et al, 1986; Swain et al, 1987; Mansi et al, 1989; Jacquillat et al, 1990; Maloisel et al, 1990; Valagussa et al, 1990; van der Wall et al, 1996). Furthermore systematic pathological assessment of chemotherapy results in the primary tumour has often been limited (Jacquillat et al, 1990; Mauriac et al, 1991) or obtained by multiple biopsies (Feldman et al, 1986; Swain et al, 1987; Mansi et al, 1989; Smith et al, 1995). Consequently, there are neither acknowledged techniques for evaluation of the response nor established prognostic variable consensus for this modality of primary management, nor any validated means to gauge its real contribution to end points as time to progression, disease-free survival (DFS) and overall survival (OS) (Fisher and Mamounas, 1995).

We present a retrospective analysis on 107 women with stage II/III non-inflammatory breast carcinoma treated between November 1980 and October 1991 with an anthracycline-based neoadjuvant chemotherapy followed by surgery. As assessment of antitumoral activity was clinical, radiological and histopathological, the study allowed comparisons of their respective value. These results, added to the 7-year median duration of follow-up, make this analysis important in identification of predictive and prognostic parameters likely to assist in the prospective evaluation design of primary cytoreduction in clinically defined localized breast cancer.

### PATIENTS AND METHODS

### Patients

Candidates to neoadjuvant chemotherapy were patients with cytologically or histologically confirmed stage I/III (UICC) breast carcinoma. Premenopausal and perimenopausal women, defined respectively by regular menstrual cycles and amenorrhoea for less than 1 year, were included in the same group of patients for the purposes of this analysis. Informed consent was obtained in all cases according to institutional guidelines.

### Pretreatment evaluation

Patients had a detailed clinical history and physical examination. Diagnosis was usually performed on fine-needle aspiration cytology. Whenever possible, steroïd hormone receptor status (oestrogen receptor, ER and progesterone receptor, PR) was established. Cytoprognostic grade was determined on an accepted and validated correspondence with the Scarff, Bloom and Richardson histopathological grading (De Maublanc, 1991). Absence of clinical metastasis was ascertained through a systematic work-up consisting of chest roentgenogram, bone scan and liver ultrasonography. Bilateral mammography completed clinical staging. Electrocardiogram, haemogram and blood biochemistry with liver function tests were performed before starting induction chemotherapy.

### Treatment

Neoadjuvant chemotherapy was based on an AVCF-type regimen (Rouëssé et al, 1986): doxorubicin 40 mg $m^{-2}$ day 1, vindesine 2 mg $m^{-2}$ day 2, cyclophosphamide 350 mg $m^{-2}$ $day^{-1}$ and fluorouracil 400 mg $m^{-3}$ day $^{-1}$ by short (20–60 min) i.v. in fusion from day 1 to day 4. Folinic acid 150 mg $m^{-2}$ day $^{-1}$ preceeded the fluorouracil administration in 50% of patients. After 1986, doxorubicin was replaced by an analogue: pirarubicin (Théprubicine, Bellon, Neuilly/Seine, France) 20 mg $m^{-2}$ $day^{-1}$ given day 1 to day 3. In absence of progression, patients were given chemotherapy every 4 weeks until no additional objective decrease in clinical size of tumour or nodes occurred. The protocol planned a maximum amount of six cycles before surgery. In case of World Health Organisation (WHO) grade III/IV extra-haematological side-effects (Miller et al, 1981), chemotherapy had to be discontinued and patients had to undergo surgery.

After primary chemotherapy completion, the choice of surgical procedure [modified radical mastectomy (MRM) or partial mastectomy] was decided individually by the collaborating surgeon. Homolateral axillary lymph node dissection was required. If breast-conservation surgical procedure was chosen, frozen-section examination informed of resection margins to provide 2-cm clearance and to remain cosmetically acceptable. If impossible on account of the width of the excision required, mastectomy was chosen.

Post-operative treatment was the responsibility of the individual, participating oncologists and there were no imposed guidelines. However, generally post-menopausal women received adjuvant tamoxifen for 2 years, while premenopausal patients with ER+ were subjected to hormonal (LHRH agonists) or surgical castration. Depending on the number of courses administered during primary chemotherapy, two or three cycles of the same combination could be given as adjuvant treatment, mainly in responding patients with positive lymph nodes. The decision to undertake locoregional radiotherapy (remaining breast and lymph nodes areas) was left to the responsible oncologist.

### Evaluation of response

Clinical patients' status was assessed at each chemotherapy session and before surgery as specified by WHO guidelines (Miller et al, 1981). Mammography was performed at the beginning and at the end of induction treatment. A partial remission (PR) implied a greater than 50% reduction of the product of the largest perpendicular diameters of measurable lesions, without the appearance of new lesions. Complete response (CR) was defined as complete disappearance of the initial tumour mass. Patients not fulfilling the criteria for CR or PR and without evidence of increase in tumour size or new areas of involvement had stable disease (SD).

We described histopathological response according to the Postsurgical Treatment Pathologic Classification given by the UICC (Beahrs et al, 1993), distinguishing pathological complete response (pCR) in primary (pTO) and lymph nodes (pNO) from other responses (pT+ or pT1–pT4, pN+). However, in some cases, we could not classify response in the primary tumour within the categories of the above-mentioned guidelines as their specimens showed residual tumour consisting of persistance of more or less diffuse scattered tumour cells microfoci with massive tumoral necrosis and areas of fibrosis. We coded such reports as pT9 in our database.

### Follow-up study

After completion of all treatment, patients were carefully followed up every 3 months for 3 years, every 6 months during the next 2 years, then at least yearly. Work-up including mammography and chest radiographs was carried out every 6 months at the beginning, then once a year. Sites of initial tumour relapse were classified as locoregional (whether exclusive or associated to distant recurrence) and distant (isolated and mixed). The database update was closed on 30 June 1994, at which time all living patients had been seen during the previous year.

### Method of analysis

Correlations between histopathological response, recurrence and main patient and tumoral characteristics were assessed with the Pearson Chi-square test. Estimates for local, metastatic and overall DFS, and OS were calculated from the date of diagnosis using the method of Kaplan and Meier (Kaplan and Meier, 1958). Univariate comparisons of end points were made with the log-rank statistic (Mantel, 1966), and a Cox survival model (Cox, 1972) was used to estimate the hazard ratio of events.

© Cancer Research Campaign 1997 

**Table 1** Pretreatment patient characteristics (n = 107)

| Characteristics | No. of patients (%) |
|---|---|
| Median age (years)(range) | 52 (28–78) |
| Menopausal status | |
| Premenopausal[a] | 50 (46.7) |
| Post-menopausal | 57 (53.3) |
| Clinical size of tumour | |
| T1 | 3 (2.8) |
| T2 | 51 (47.7) |
| T3 | 44 (41.1) |
| T4 | 8 (7.5) |
| Tx | 1 (0.9) |
| Clinical lymph node status | |
| N0 | 60 (56.1) |
| N1 | 37 (34.6) |
| N2 | 10 (9.3) |
| Clinical stage of the disease | |
| IIA | 39 (36.4) |
| IIB | 33 (30.8) |
| IIIA | 26 (24.3) |
| IIIB | 8 (7.5) |
| X | 1 (0.9) |
| Cytoprognostic grade | |
| 1 | 14 (13.1) |
| 2 | 43 (40.2) |
| 3 | 33 (30.8) |
| Not available | 17 (15.9) |
| Hormonal receptor status | |
| $ER^-PR^-$ | 32 (29.9) |
| $ER^-PR^+$ | 4 (3.7) |
| $ER^+PR^+$ | 38 (35.5) |
| $ER^+PR^-$ | 18 (16.8) |
| Not available | 15 (14.0) |

[a]Including five perimenopausal patients.

**Table 2** Distribution of patient characteristics according to different histopathological responses in the primary tumour (n = 105)

| Characteristic | pT0 | pT1–pT3 | pT9[a] | Total |
|---|---|---|---|---|
| No. of patients | 11 | 76 | 18 | 105[b] |
| Menopausal status | | | | |
| Premenopausal | 5 | 35 | 10 | 50 |
| Post-menopausal | 6 | 41 | 8 | 55 |
| Initial clinical T | | | | |
| T1 | 0 | 1 | 2 | 3 |
| T2 | 9 | 39 | 2 | 50 |
| T3 | 1 | 30 | 13 | 44 |
| T4 | 0 | 6 | 1 | 7 |
| Tx | 1 | 0 | 0 | 1 |
| Initial clinical N | | | | |
| N0 | 11 | 40 | 8 | 59 |
| N1 | 0 | 28 | 8 | 36 |
| N2 | 0 | 8 | 2 | 10 |
| Initial clinical stage | | | | |
| IIA | 9 | 27 | 3 | 39 |
| IIB | 1 | 24 | 7 | 32 |
| III | 0 | 25 | 8 | 33 |
| X | 1 | 0 | 0 | 1 |
| Cytoprognostic grade | | | | |
| 1 | 1 | 12 | 1 | 14 |
| 2 | 6 | 31 | 4 | 41 |
| 3 | 1 | 22 | 10 | 33 |
| Not available | 3 | 11 | 3 | 17 |
| Oestrogen receptor status | | | | |
| $ER^-$ | 4 | 23 | 9 | 36 |
| $ER^+$ | 4 | 46 | 5 | 55 |
| Not available | 3 | 7 | 4 | 14 |
| Clinical response | | | | |
| Complete | 6 | 11 | 8 | 25 |
| Partial | 5 | 52 | 9 | 66 |
| Stable | 0 | 11 | 1 | 12 |
| Not available | 0 | 2 | 0 | 2 |
| Radiological response | | | | |
| Complete | 2 | 0 | 2 | 4 |
| Partial | 3 | 40 | 10 | 53 |
| Stable | 1 | 22 | 2 | 25 |
| Not available | 5 | 14 | 4 | 23 |
| Pathological N | | | | |
| pN0 | 11 | 41 | 11 | 63 |
| pN+ | 0 | 35 | 7 | 42 |

[a]pT9, pathological response not able to be classed within categories of the Postsurgical Treatment Pathologic Classification given by the UICC (Beahrs et al, 1993) and consisting of tumoral necrosis, areas of fibrosis with persistance of microscopic nests and/or diffuse scattered tumour cells foci. [b]Pathological tumour size assessment lacking in two patients (i.e. pTx).

### RESULTS

### Patient characteristics

From November 1980 to October 1991, 125 women were entered in this study, of whom only three had stage I tumours and eight presented inflammatory breast cancer. These patients were excluded from analysis on account of their different natural history and prognosis (Henderson, 1991), in addition to seven other patients refusing surgery after induction chemotherapy. Thus, of 125 patients, 107 (86%) with stage II/III non-inflammatory breast carcinoma and who underwent primary chemotherapy plus surgery are considered in this report. Table 1 summarizes their main characteristics.

### Treatment and toxicity

The median number of cycles of induction chemotherapy administered every 4 weeks per patient was six (range two to six), without dose reduction in > 90% of patients. Except in cases in which patients developed severe extra haematological toxicity (24 patients), surgery was done at the maximal clinical response recording time, i.e. after 2, 3, 4, 5 and 6 cycles in 1, 11 (10.3%), 19 (17.8%), 17 (15.9%) and 59 (55.1%) patients respectively. Grade III/IV myelosuppression complicated with fever requiring hospitalization was seen in 15 patients (14%), two patients developed grade III acral paraesthesias to vindesine, and asymptomatic decline in left ventricular ejection fraction in three patients motivated cessation of chemotherapy after five cycles. Digestive tolerance was good except in four patients who presented severe vomiting (grade IV). Despite application of refrigerated cap, alopecia of some degree was observed in most patients. Partial mastectomy was the chosen surgical procedure in 37 patients (34.6%) while 70 (65.4%) underwent MRM. After surgery, all patients were free of disease.

Adjuvant treatment included hormonotherapy with tamoxifen in 47 of 57 post-menopausal women (82.5%). Seventeen of

© Cancer Research Campaign 1997

**Table 3** Disease outcome in patients with stage II/III breast cancer (univariate analysis)

| Variable | n | LRFa (%) | P-value | Mb (%) | P-value | Deaths (%) | P-value |
|---|---|---|---|---|---|---|---|
| Clinical T | | | | | | | |
| T1/T2 | 54 | 10 (18.5) | | 5 (09.3) | | 8 (14.8) | |
| T3/T4 | 52 | 20 (38.5) | **0.008** | 22 (42.3) | **0.00001** | 23 (44.2) | **0.001** |
| Clinical N | | | | | | | |
| N0 | 60 | 9 (15.0) | | 7 (11.7) | | 6 (10.0) | |
| N1/N2 | 47 | 21 (44.7) | **0.00001** | 20 (42.6) | **0.00001** | 25 (53.2) | **0.00001** |
| Clinical stage | | | | | | | |
| IIA | 39 | 5 (12.8) | | 3 (07.7) | | 4 (10.3) | |
| IIB | 33 | 8 (24.2) | | 7 (21.2) | | 8 (24.2) | |
| III | 34 | 17 (50.0) | **0.0002** | 17 (50.0) | **0.00001** | 19 (55.9) | **0.00001** |
| Oestrogen receptor | | | | | | | |
| $ER^+$ | 56 | 13 (23.2) | | 11 (19.6) | | 12 (21.4) | |
| $ER^-$ | 36 | 10 (27.8) | >0.5 | 12 (33.3) | 0.078 | 16 (44.4) | **0.02** |
| Cytoprognostic grade | | | | | | | |
| 1–2 | 57 | 7 (12.3) | | 10 (17.5) | | 10 (17.5) | |
| 3 | 33 | 15 (45.5) | **0.0001** | 10 (30.3) | **0.039** | 16 (48.5) | **0.0004** |
| Clinical response | | | | | | | |
| CR | 26 | 7 (26.9) | | 4 (15.4) | | 5 (19.2) | |
| PR | 67 | 19 (28.4) | | 19 (28.4) | | 22 (32.8) | |
| SD | 12 | 4 (33.3) | >0.5 | 4 (33.3) | >0.5 | 4 (33.3) | >0.5 |
| Time to clinical response/Cc | | | | | | | |
| ≤ C3 | 68 | 19 (27.9) | | 17 (25.0) | | 20 (29.4) | |
| > C3 | 25 | 7 (28.0) | >0.5 | 6 (24.0) | >0.5 | 7 (28.0) | >0.5 |
| Pathological T | | | | | | | |
| pT0 | 11 | 0 (0.00) | | 0 (0.00) | | 0 (0.00) | |
| pT1–3 | 76 | 19 (25.0) | | 21 (27.6) | | 22 (28.9) | |
| pT9 | 18 | 11 (61.1) | **0.0009** | 6 (33.3) | 0.19 | 9 (50.0) | 0.099 |
| Pathological N | | | | | | | |
| pN0 | 63 | 11 (17.5) | | 10 (15.9) | | 8 (12.7) | |
| pN+ | 42 | 18 (42.9) | **0.0008** | 17 (40.5) | **0.0008** | 22 (52.4) | **0.00001** |

[a]LRF, locoregional failures (whether exclusive or associated to distant recurrence). [b]M, distant metastasis (whether exclusive or associated to locoregional recurrence). [c]C, cycle.

twenty-four (70.8%) premenopausal patients with ER+ underwent castration. Locoregional irradiation was carried out in 35 patients; in 32 of 96 (33.3%) patients showing viable tumoral cells on the surgical specimen after either conservative surgery [16/32 women (50%] or MRM [16/64 (25%)] and in 3 of 11 patients with a pCR. In patients receiving radiotherapy, no chest wall infection or moist desquamation was observed. Adjuvant chemotherapy was delivered to 30 women; in 14 of 33 (42.4%) women with cytoprognostic grade 3 tumours, in 13 of 36 (36.1%) with ER– and in 23 of 42 (54.8%) with pN+. In all, adjuvant hormonotherapy was given to 77 patients (72%) and, in 17 instances, with concurrent chemotherapy.

### Clinical and radiological response

No tumoral progression occurred during chemotherapy. In 26 patients (24.3%), a clinical CR was documented, whereas 67 patients (62.6%) presented a PR. This clinical response occurred before the third cycle in 68 patients (64%). Stable disease was observed in 12 patients (11.2%) (data lacking for two patients).

Radiological response assessment was available in 84 women. Among them, five (6%) were classified as CR, whereas PR was recorded in 54 others (64.3%). Of 18 patients with clinical CR and mammographic assessment available, only five (27.8%) showed a simultaneous radiological CR, illustrating the lack of correlation between radiological and clinical evaluations.

Clinical and radiological responses were not related to menopausal and ER status nor to cytoprognostic grade (data not shown). There was also no significant difference according to the anthracyclin used or initial TNM. Among 54 T1/T2 tumours, 17 (31.5%) achieved clinical CR and 28 (51.9%) PR, while CR and PR were documented respectively in 9 (17.3%) and 38 (73.1%) of the 52 T3/T4 tumours ($P > 0.5$).

### Patholological response (Table 2)

Histopathological response assessment in both the primary (pT) and lymph nodes (pN) was available in 105 of 107 (98.1%) patients (data incomplete in two patients). Ductal carcinoma in situ alone was never seen, whereas 19 specimens showed coexistence of residual invasive tumour (pT+) and small foci of intraductal carcinoma.

We observed 11 pCR (10.3%) in the primary tumour (pTO), in all cases without microscopically involved lymph nodes (pNO).

© Cancer Research Campaign 1997

**Figure 1** Disease-free survival according to initial clinical stage (median follow-up 81 months). The differences between the three curves are statistically significant (log-rank P = 0.00001). Straight sticks represent patients at risk. Numbers in brackets are patients remaining at risk at the end of the curves

**Figure 2** Overall survival according to histological axillary node dissection results (median follow-up 81 months). The difference between the curves of the two groups of women, with or without lymph nodes involvement, is statistically significant (log-rank P = 0.00001). Straight sticks represent patients at risk. Numbers in brackets are patients remaining at risk at the end of the curves

Six of those eleven patients had been assessed as clinical CR, five had been clinically classified as PR, only two showing radiological CR. Initial clinical T and N were significantly correlated to the attainment of a pTO status ($P \leq .005$), which was observed mostly in early stage tumours (IIA), all with initial NO disease.

In 18 patients, pathological assessment could not be classified within the pT categories according to the Postsurgical Treatment Pathological Classification. Their residual tumour histopathological pattern was coded as pT9 (see Patients and methods). Among them, we documented 11 pNO (61.1%) (Table 2). In this small sample, pT9 pathological assessment was found to be unrelated to any factor other than large tumours (T3).

Of 60 women with clinical NO, 13 (21.7%) had pN+ after primary chemotherapy (11 of them with more than three involved nodes). Of 37 women with N1 disease, 21 (56.8%) had pN+. Eight of ten patients with N2 disease had pN+. Overall, 15 patients (14%) had more than three involved nodes. The total of women with pNO was 63 (58.9%).

When logistic regression analysis was performed with pretherapy patient characteristics, clinical and radiological responses, only clinical stage IIA appeared significantly correlated with either pNO ($P = 0.0001$) or pTO ($P = 0.004$). Of note, there was no correlation between pathological response and clinical or radiological response.

**Table 4** Multivariate analysis according to Cox model

| End points | Significant prognostic factors[a] | P-value | Relative risk | 95% CI |
|---|---|---|---|---|
| Cumulative incidence of LRF[b] | | | | |
| | Cytoperognostic grade (1–2/3) | 0.014 | 3.7 | 1.3–10.7 |
| | pT (pTO/pT1–pT3/pT9) | 0.004 | 4.6 | 1.6–13.2 |
| | pN (pNO/pN+) | 0.0008 | 7.7 | 2.3–25.6 |
| Cumulative incidence of M[c] | | | | |
| | Cytoprognostic grade (1–2/3) | 0.06 | 2.6 | 0.9–7.10 |
| | T (T1–T2/T3–T4) | 0.002 | 7.6 | 2–28.0 |
| | N (NO/N1–N2) | 0.009 | 4.5 | 1.5–13.8 |
| DFS | | | | |
| | Cytoprognostic grade (1–2/3) | 0.009 | 2.8 | 1.2–6.03 |
| | T (T1–T2/T3–T4) | 0.014 | 11.5 | 1.6–80.5 |
| | N (NO/N1–N2) | 0.008 | 5.4 | 1.5–18.7 |
| | pN (pNO/pN+) | 0.004 | 4.6 | 1.6–13.1 |
| OS | | | | |
| | Cytoprognostic grade (1–2/3) | 0.005 | 4.1 | 1.5–10.8 |
| | T (T1–T2/T3–T4) | 0.012 | 22.4 | 1.9–256 |
| | N (NO/N1–N2) | 0.001 | 15.2 | 2.9–77.7 |
| | pN (pNO/pN+) | 0.034 | 5.4 | 1.1–25.4 |

[a]Not significant prognostic factors (1) for cumulative incidence of LRF: ER status, T, N and stage; (2) for cumulative incidence of M: ER status, stage, pT and pN; (3) for DFS and OS: ER status, stage and pT. [b]LRF, locoregional failures (whether exclusive or associated to distant recurrence). [c]M, distant metastasis (whether exclusive or associated to locoregional recurrence).

 © Cancer Research Campaign 1997

### Tumour recurrence and survival

With a median follow-up of 81 months (range 32–164+ months), we have observed 46 relapses (43%). Local relapse alone occurred in 19 women (17.8%) (including two patients who developed contralateral breast cancer for the purposes of the analysis); 16 (15%) developed distant metastasis as the first sign of disease relapse, while synchronous detection of distant metastasis and locoregional failure was seen in 11 patients (10.3%). Thirty-one patients (29%) have died of metastatic breast cancer. We registered no other causes of death. The median DFS for the cohort is 90.5 months, while median OS has not been yet reached.

### Univariate analysis

Recurrence and its type and death rate were significantly related to well-established variables such as T, N, stage and cytoprognostic grade (Table 3), unlike menopausal status (data not shown). ER status showed a significant correlation only with survival ($P$ = 0.02). Of note, clinical response (as well as radiological) and time to clinical response did not reach a statistically significant value on failure rate. Relapse and OS were significantly related to pN (Table 3). Among 42 patients with pN+, 17 (40.5%) developed metastasis and 20 (47.6%) are still alive, compared with 10 (15.9%) metastatic relapses and eight (12.7%) deaths in 63 women with pNO ($P < 0.001$).

Locoregional failure was significantly correlated to pT, being unfavourable to the 18 patients with a pT9 assessment: 11 (61.1%) developed local relapse compared with only 19 of, 76 (25%) with pT1–pT3 response ($P$ = 0.0009). Although pT failed to significantly correlate with metastasis likelihood or survival (Table 3), all pTOpNO patients remain free of recurrence with a median observation time of 68 months (range 38–148+ months). One of them developed ovarian cancer after 130 months of follow-up. Presence of intraductal carcinoma was not associated with incidence of local recurrence; of 19 patients with this histological pattern plus residual invasive tumour (pT+) on the surgical specimen, five developed local relapse (26.3%) compared with 23 in the 77 other pT+ patients (29.9%) without any intraductal component ($P > 0.5$).

The type of surgery and adjuvant radiotherapy does not seem to have influenced locoregional recurrence rate. Of 32 pT+ patients after conservative surgery, 16 only received local radiotherapy. However, the rate of local relapse was the same whether they had received it or not (5 of 16 in both groups, 31.3%). The same remark is valid for adjuvant radiotherapy in the group of pT+ patients after MRM (data not shown). On the other hand, the ones who underwent radiotherapy in this group had more often more than three involved nodes (7 of 16 patients) compared with the others (6 of 48). In patients who received adjuvant chemotherapy (30 women), 12 (40%) developed distant metastasis compared with 15 in 77 (19.5%) without post-surgical chemotherapy. Within these two groups of patients, the former showed a relative high frequency of tumours with ER– (43.3%), cytoprognostic grade 3 (46.7%) and/or pN+ (76.7%) compared with the second (respective rates of 29.9%, 24.7% and 26%). The same proportion of distant metastasis was seen in women receiving adjuvant hormonotherapy (19 of 77 patients) or not (8 of 30).

In patients having undergone breast conservation procedure initially, total mastectomy seemed the mainstay treatment for locoregional relapse alone (8 of 17 patients, not including the two patients with contralateral breast cancer). All had salvage mastectomy and radiotherapy was performed in one patient. Five patients remain free of second failure, while three have developed distant metastasis, their median second relapse-free interval being 15 months (range 2–44+ months). After MRM and in case of local failure (9 of 17 patients), treatment consisted of either surgery (two patients), radiotherapy (three patients) or a combination of radiotherapy and surgery (three patients). One patient refused any treatment and died later of metastatic disease. This salvage treatment prevented later recurrence in only one of these women, the others developing relapse with a median interval of 6 months (range 2–50 months).

Figure 1 shows the actuarial DFS according to clinical staging. Both the 5-and 10-year DFS rates are 81% for 39 stage IIA tumours, whereas the 5-year DFS rate is 61% for 33 patients with stage IIB tumours. Women with stage III tumours (34 patients) have a 5-year DFS rate of 40%, and their median value for DFS is 33 months. The differences between the three curves are statistically significant ($P$ = 0.00001). In Figure 2, survival according pN status shows 5- and 10-year OS rates of 92% and 75%, respectively, in patients with pNO, while for those with either ≤ 3 or > 3 involved nodes actuarial OS curves meet at 54 months, 5-year OS rate is 54% and median values for OS reach about 70 months ($P$ = 0.00001).

### Multivariate analysis\

As shown in Table 4, we failed to correlate ER status and clinical stage with either locoregional failure rate, metastasis rate, DFS or OS. Multivariate analysis preserved the significance of cytoprognostic grade to the four above mentioned end points. Both pT and pN were significantly correlated with locoregional failure, while only pN contributed further information to DFS and OS. Clinical T and N were the other significant factors associated with incidence of metastasis, DFS and OS.

### DISCUSSION

Although primary chemotherapy limits the possibility to study the initial biological characteristics of the tumour, it has gained popularity in locally advanced breast cancer in the past 15 years, increasing potential for breast preservation (Fisher and Mamounas, 1995). During this time, adjuvant chemotherapy proved itself gradually (EBCTCG, 1992), and several groups chose to deliver it in neoadjuvant setting for earlier stages (Jacquillat et al, 1990; Fisher et al, 1994; Schwartz et al, 1994; Semiglazov et al, 1994). Our group has been involved in this strategy since 1980, resorting to an anthracycline-based regimen, in agreement with its initial experience which favours their use in adjuvant setting for premenopausal patients with pN+ (Misset et al, 1996). After 1986, the search for new anthracyclines less cardiotoxic than doxorubicin resulted, in France, in the availability of pirarubicin, which was chosen according to encouraging early results (Chevallier et al, 1992).

Our results on survival for stage II/III tumours are similar to data from published series of preoperative chemotherapy (Hortobagyi et al, 1988; Jacquillat et al, 1990; Schwartz et al, 1994). Furthermore, the value of DFS and OS for patients with stage II tumours seems to favour the use of neoadjuvant chemotherapy, with at least a delay in disease progression parameters, when compared with historical experiences of post-operative chemotherapy for primary operated patients (i.e. patients with early breast cancer). The 5- and 10-year DFS rates are both 81% for stage IIA, the 5-year rate is 61% for stage IIB, which compares

© Cancer Research Campaign 1997

favourably with the outcome of the first CMF adjuvant programme of the Milan Cancer Institute (Bonadonna, 1992). Nevertheless, this comparison must be interpreted cautiously as the exact initial pN was not known in our study, while all women included in the Italian trial were pN+. Moreover, only a few reports have addressed the role of neoadjuvant chemotherapy in stage II breast cancer (67% of our patients' population) (Jacquillat et al, 1990; Schwartz et al, 1994; Semiglazov et al, 1994).

Recognition of histopathological response as a significant parameter in determining long-term prognosis of women submitted to primary chemotherapy appears to be the most important contribution of the present report, strengthening the conclusions of several authors (Feldman et al, 1986; Hortobagyi et al, 1988; Maloisel et al, 1990; Ragaz et al, 1994; Frye et al, 1995). Obtaining a simultaneous pCR in primary and lymph nodes seems a valid surrogate end point for long-term relapse-free status. Although pT did not reach a significant value on DFS in multivariate analysis, none of the 11 women who achieved pTOpNO status has relapsed with a median follow-up of 5.7 years. They did not show a specific pretreatment characteristics distribution other than clinical stage, all but two being stage IIA. The British Columbia Cancer Agency reported 16 pCR (21.6%) obtained in 74 stage III tumour patients after a 6-month ACMF induction regimen (Ragaz et al, 1994). Among these 16 patients, they observed four relapses (25%). Nevertheless, the significant contribution to survival of pCR remains considerable in these locally advanced tumours as 60% of the 58 remaining patients developed recurrence. These results are reinforced by a Russian randomized trial comparing neoadjuvant chemotherapy combined to radiotherapy with radiotherapy alone before mastectomy in 271 patients with stage IIB/IIIA breast cancer (Semiglazov et al, 1994). In the arm with both induction treatments, they observed 30% pCR, 95% remaining free of relapse with a median follow-up time of 53 months. However, both studies report induction chemotherapy combined to radiotherapy, which makes it difficult to elicit the respective roles of both modalities in obtaining a pCR.

Our data also indicate that one should consider carefully the method used for the pathological response evaluation. Multiple biopsies or fine-needle aspirations as done by several investigators (Feldman et al, 1986; Swain et al, 1987; Briffod et al, 1989; Mansi et al, 1989; Smith et al, 1995) may be insufficient. It could preclude identification of a clinical subpopulation with, in particular, a high cumulative incidence of locoregional relapse, which is reflected in our multivariate analysis giving high significance to pT for locoregional failure rate ($P$ = 0.004). Thus, in 18 patients (classified as pT9), despite significant chemosensitivity pathological marks, we observed residual scattered tumoral cells microfoci, which may reveal a contingent of tumoral cells resistant to chemotherapy. Of these patients, 60% developed local recurrence, and 50% have died from metastatic breast cancer, even when pNO was documented. This last point is noteworthy as pNO was a highly significant predictive factor for long DFS and OS both in univariate and multivariate analysis. The assessment of residual microscopic disease might erase the benefit in obtaining pNO, and the incorporation of these pT9 patients to the group with putatively beneficial histopathological downstaging, as advocated by others (Smith et al, 1995), may still be arguable. Although the impact of axillary lymph node dissection on survival is often questioned in primary operated patients (Lin et al, 1993), our study emphasizes its major prognostic information for women submitted to primary chemotherapy, as in the last report from the MD Anderson Hospital (Frye et al, 1995). On the other hand, presence of an intraductal component with residual invasive tumour was not a predictor of local relapse, contrasting with conclusions of others (Schnitt et al, 1984). In this last report, patients had earlier stage tumours, and treatment consisted of local excision plus radiotherapy, which may explain the different observations.

Non-invasive methods for response evaluation (clinical and radiological measurements) did not show any significant prognostic value on patient outcome. However, the only published report (Jacquillat et al, 1990) showing improved DFS for patients who had a major clinical tumour regression following primary chemotherapy cannot be compared with our study as locoregional treatment excluded surgery. Correlation between clinical and radiological response were partial and inaccurate. Like other investigators (Feldman et al, 1986; Fisher et al, 1994; van der Wall et al, 1996), we failed to establish any correlation between any of these two response evaluation methods and pT or pN. This incites us to warn against the exclusive use of both these techniques for evaluation of the tumour response to primary chemotherapy.

In conclusion, the timing of chemotherapy for non-metastatic breast cancer is still debated, and results of a randomized NSABP B-18 trial comparing four cycles of cyclophosphamide and doxorubicin before or after surgery are eagerly awaited (Fisher et al, 1994). However, our study supports that a pCR in both the primary tumour and lymph nodes has a major prognostic value in patients being treated with neoadjuvant chemotherapy for breast cancer. The role of surgery of the primary tumour and lymph nodes as an evaluation tool should be considered before further assumptions are made of its exclusion in programmes of primary chemotherapy for breast cancer.

### REFERENCES

Beahrs OH, Henson DE, Hutter RVP and Kennedy BJ (eds) (1993) *Manual for Staging of Cancer*, 4th edn, pp. 161–167. JB Lippincott: Philadelphia, PA

Bonadonna G (1992) Evolving concepts in the systemic adjuvant therapy of breast cancer. *Cancer Res*, **52**: 2127–2137

Bonadonna G, Veronesi U, Brambilla C, Ferrari L, Luini A, Greco M, Bartoli C, Coopmans De Yoldi G, Zucali R, Rilke F, Amdreola S, Silvestrini R, Di Fronzo G and Valagussa P (1990) Primary chemotherapy to avoid mastectomy in tumors with diameters of three centimeters or more. *J Natl Cancer Inst* **82**: 1539–1545

Briffod M, Spyratos F, Tubiana-Hulin M, Pallud C, Mayras C, Filleul A and Rouëssé J (1989) Sequential cytopunctures during preoperative chemotherapy for primary breast carcinoma. *Cancer* **63**: 631–637

Chevallier B, Mignot L, Delozier T, Morvan F, Ferme C and Herait P (1992) Phase II study of tetrahydropyranyl adriamycin (pirarubicin) in elderly patients with advanced breast cancer. *Am J Clin Oncol (CCT)* **15**: 395–398

Cox DR (1972) Regression models and life-tables. *J R Stat Soc [B]* **34**: 187–220

De Lena M, Zucali R, Viganotti G, Valagussa P and Bonadonna G (1978) Combined chemotherapy–radiotherapy approach in locally advanced (T3b-T4) breast cancer. *Cancer Chemother Pharmacol* **1**: 53–59

De Maublanc MA (1991) Les données récentes de la cytoponction mammaire. *Ann Pathol* **11**: 299–308

Early Breast Cancer Trialists' Collaborative Group (1992) Systemic treatment of early breast cancer by hormonal, cytotoxic, or immune therapy: 133 randomised trials involving 31 000 recurrences and 24 000 deaths among 75 000 women. *Lancet* **339**: 1–15, 71–84

Feldman LD, Hortobagyi GN, Buzdar AU, Ames FC and Blumenshein GR (1986) Pathological assessment of response to induction chemotherapy in breast cancer. *Cancer Res* **46**: 2578–2581

Fisher B and Mamounas EP (1995) Preoperative chemotherapy: a model for studying the biology and therapy of primary breast cancer. *J Clin Oncol* **13**: 537–540

Fisher B, Rockette H, Robidoux A, Margolese R, Cruz A, Hoehn J, Boysen D, Mamounas E, Wickerham DL and Decillis A (1994) Effect of preoperative

 © Cancer Research Campaign 1997

therapy for breast cancer (BC) on local-regional disease: first report of NSABP B-18 (abstract 57). *Proc Am Soc Clin Oncol* **13**: 65

Fletcher G (1972) Local results of irradiation in the primary management of localized breast cancer. *Cancer* **29**: 545–551

Frye D, Buzdar A and Hortobagyi G (1995) Prognostic significance of axillary nodal involvement after preoperative chemotherapy in stage III breast cancer (abstract 81). *Proc Am Soc Clin Oncol* **14**: 95

Henderson IC (1991) Prognostic factors. In *Breast Diseases*, 4th edn, Harris JR, Hellman S, Henderson IC and Kinne DW. (eds), pp. 332–346. JB Lippincott: Philadelphia, PA

Hortobagyi GN, Ames FC, Buzdar AU, Kau SW, McNeese MD, Paulus D, Hug V, Holmes FA, Romsdahl MM, Fraschini G, McBride CM, Martin RG and Montague E (1988) Management of stage III primary breast cancer with primary chemotherapy, surgery, and radiation therapy. *Cancer* **62**: 2507–2516

Jacquillat C, Weil M, Baillet F, Borel C, Auclerc G, De Maublanc MA, Housset M, Forget G, Thill L, Soubrane C and Khayat D (1990) Results of neoadjuvant chemotherapy and radiation therapy in the breast-conserving treatment of 250 patients with all stages of infiltrative breast cancer. *Cancer* **66**: 119–129

Kaplan EL and Meier P (1958) Nonparametric estimation from incomplete observations. *J Am Sta Assoc* **53**: 185, 1457–1481

Lin PP, Allison DC, Wainstock J, Miller KD, Dooley WC, Friedman N and Baker RR (1993) Impact of axillary lymph node dissection on the therapy of breast cancer. *J Clin Oncol* **11**: 1536–1544

Maloisel F, Dufour P, Bergerat JP, Herbrecht R, Duclos B, Boilletot A, Giron C, Jaeck D, Haennel P, Jung G and Oberling F (1990) Results of initial doxorubicin, 5-fluorouracil and cyclophosphamide combination chemotherapy for inflammatory carcinoma of the breast. *Cancer* **65**: 851–855

Mansi JL, Smith IE, Walsh G, A'Hern RP, Harmer CL, Sinnett HD, Trott PA, Fisher C and McKinna JA (1989) Primary medical therapy for operable breast cancer. *Eur J Cancer Clin Oncol* **25**: 1623–1627

Mantel N (1966) Evaluation of survival data and two row rank order statistics arising in its consideration. *Cancer Chemother Rep* **50**: 163–170

Mauriac L, Durand M, Avril A and Dilhuydy JM (1991) Effects of primary chemotherapy in conservative treatment of breast cancer patients with operable tumors larger than 3 cm. *Ann Oncol* **2**: 347–354

Miller A, Hoogstraten B, Staquet M and Winkler P (1981) Reporting results of cancer treatment. *Cancer* **47**: 207–214

Misset J-L, DI Palma M, Delgado M, Plagne R, Chollet P, Fumoleau P, Le Mevel B, Belpomme D, Guerrin J, Fargeot P, Metz R, Ithzaki M, Hill C and Mathe G (1996) Adjuvant treatment of node-positive breast cancer with cyclophosphamide, doxorubicin, fluorouracil, and vincristine versus cyclophosphamide, methotrexate, and fluorouracil: final report after 16-year median follow-up duration. *J Clin Oncol* **14**: 1136–1145

Ragaz J, Manji M, Plenderleith IH, Rebbeck P, Baird R, Basco V, Kuusk U, Olivotto I, McFarlane J, Clay G, Brown D, Worth A, O'Reilly S, Gelmon K, McGregor G, Spinelli J and Coldman A (1994) Impact of the residual disease in stage III breast cancer patients treated with preoperative (neoadjuvant) chemotherapy (CT), radiotherapy (XRT) and mastectomy. The 10 year analysis of the British Columbia study (abstract 88). *Proc Am Soc Clin Oncol* **13**: 71

Rouëssé J, Friedman S, Sarrazin D, Mouriesse H, Le Chevalier T, Arriagada R, Spielmann M, Papacharalambous A and May-Levin F (1986) Primary chemotherapy in the treatment of inflammatory breast carcinoma: a study of 230 cases from the institut Gustave-Roussy. *J Clin Oncol* **4**: 1765–1771

Schaake-Koning C, van Der Linden EH, Hart G and Engelsman E (1985) Adjuvant chemo- and hormonal therapy in locally advanced breast cancer: a randomized clinical study. *Int J Radiation Oncol Biol Phys* **11**: 1759–1763

Schnitt SJ, Connoly JL, Harris JR, Hellman S and Cohen RB (1984) Pathologic predictors of early recurrences in stage I and II breast cancer treated by primary radiation therapy. *Cancer* **53**: 1049–1057

Schwartz GF, Birchansky CA, Komarnicky LT, Mansfield CM, Cantor RI, Biermann WA, Fellin FM and McFarlane J (1994) Induction chemotherapy followed by breast conservation for locally advanced carcinoma of the breast. *Cancer* **73**: 362–369

Semiglazov VF, Topuzov EE, Bavli JL, Moiseyenko VM, Ivanova OA, Seleznev IK, Orlov AA, Barash NY, Golubeva OM and Chepic OF (1994) Primary (neoadjuvant) chemotherapy and radiotherapy compared with primary radiotherapy alone in stage IIb-IIIa breast cancer. *Ann Oncol* **5**: 591–595

Smith LE, Walsh G, Jones A, Prendiville J, Johnston S, Gusterson B, Ramage F, Robertshaw H, Sacks N, Ebbs S, McKinna JA and Baum M (1995) High complete remission rates with primary neoadjuvant chemotherapy for large early breast cancer. *J Clin Oncol* **13**: 424–429

Swain SM, Sorace RA, Bagley CS, Danforth DN, Bader J, Wesley MN, Steinberg SM and Lippman ME (1987) Neoadjuvant chemotherapy in the combined modality approach of locally advanced non metastatic breast cancer. *Cancer Res* **47**: 3889–3894

Valagussa P, Zambetti M, Bonadonna G, Zucali R, Mezzanotte G and Veronesi U (1990) Prognostic factors in locally advanced noninflammatory breast cancer. Long-term results following primary chemotherapy. *Breast Cancer Res Treat* **15**: 137–147

Van Der Wall E, Rutgers EJT, Holtkamp MJ, Baars JW, Schornagel JH, Peterse JL, Beijnen JH and Rodenhuis S (1996) Efficacy of up-front 5-fluorouracil–epidoxorubicin–cyclophosphamide (FEC) chemotherapy with an increased dose of epidoxorubicin in high-risk breast cancer patients. *Br J Cancer* **73**: 1080–1085

Zucali R, Uslenghi C, Kenda R and Bonadonna G (1976) Natural history and survival of inoperable breast cancer treated with radiotherapy followed by radical mastectomy. *Cancer* **37**: 1422–1431

© Cancer Research Campaign 1997